# Delayed Intracerebral Hemorrhage Secondary to Ventriculoperitoneal Shunt: A Case Report and Literature Review

**DOI:** 10.1097/MD.0000000000002029

**Published:** 2015-10-30

**Authors:** Li Ma, Yi-Li Chen, Shu-Xu Yang, Yi-Rong Wang

**Affiliations:** From the Department of Neurosurgery, Sir Run Run Shaw Hospital, Zhejiang University School of Medicine, Hangzhou, Zhejiang Province, China.

## Abstract

The ventriculoperitoneal (VP) shunt is a routine procedure for cerebrospinal fluid (CSF) diversion, and is associated with many complications. A delayed hemorrhage after the VP shunt surgery, however, is quite rare. In this study, we report a case involving late-onset hemorrhage. The 67-year-old male patient with a history of head trauma and brain surgery underwent a VP shunt placement for hydrocephalus. The surgery course was uneventful and no bleeding was revealed in the first computed tomographic (CT) scan after the procedure. However, a massive intraparenchymal and intraventricular hemorrhage occurred 8 h following adjustment of the valve system on the 8th day after surgery.

Erosion of the vasculature by catheter cannulation and a sudden reduction of CSF pressure after downregulation of the valve could be one of the possible causes of the intracerebral hemorrhage (ICH).

## INTRODUCTION

Ventriculoperitoneal (VP) shunt placement is considered to be one of the most common procedures in the neurosurgery department. Infection, subdural hemorrhage, seizures, and shunt malfunction are among the most common complications secondary to VP shunt surgery, which have been extensively reported. Soon after operation, it is also not uncommon to recognize small amounts of blood in the ventricle or in the parenchyma around the tubing.^1^ In some cases, no bleeding is observed in the cerebrospinal fluid when performing ventricular cannulation or in the CT/magnetic resonance imaging (MRI) scan right after surgery; however, intraparenchymal hemorrhage can be detected on follow-up images. Delayed intracerebral hemorrhage after the placement of a VP shunt (DIHAV) is very rare. DIHAV was first described by Matsumura et al in 1985. Now there are approximately only 12 cases that have been revealed in the literature (Table 1). The mortality of DIHAV is as high as 50%, whereas the underlying mechanisms are not fully elucidated. In the current report, we present a typical case with extensive intraparenchymal and intraventricular hemorrhage 8 days after a VP shunt placement. We also discuss the potential mechanisms that are involved and review relevant literature.

**TABLE 1:**
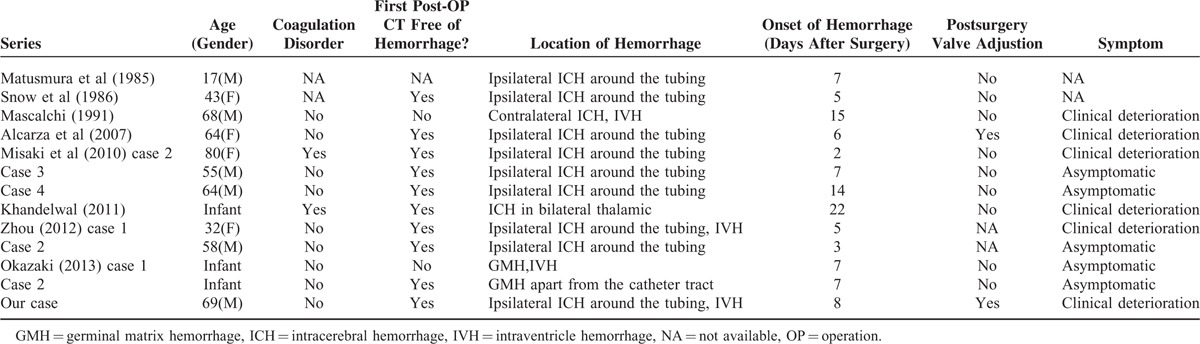
Literature Review of Cases of Delayed Intracerebral Hemorrhage After Ventriculoperitoneal Shunt Placement

## CASE REPORT

A 67-year-old male was admitted to the emergency department of our hospital due to severe head trauma. The patient was in a coma with a Glasgow Coma Scale (GCS) score of 9 upon admission. A head CT scan revealed a bilateral frontal contusion and a large left frontal lobe hemorrhage. A left cranioectomy and hematoma evacuation was performed and the postoperation clinical situation kept stable. Eight weeks later, the patient underwent another surgery for cranioplasty and was then discharged for further rehabilitation.

**FIGURE 1:**
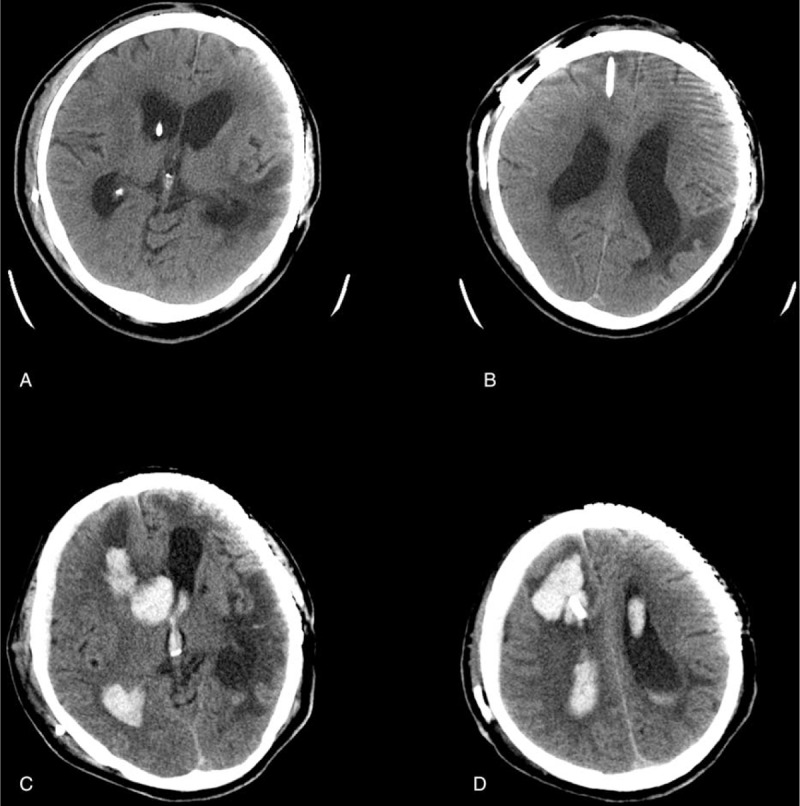
CT scans of pre- and posthemorrhage onset of the patient. (A, B) CT scan on 8th day after VP shunt showed enlarged ventricles without any bleeding. (C, D) CT scan after clinical deterioration revealed a massive intraparenchymal and intraventricular hematoma along the path of ventricular catheter. CT = computed tomographic, VP = ventriculoperitoneal.

During the next couple of months, the patient initially did quite well. He could walk without assistance and was able to take care of his own affairs. However, 10 months after the first surgery, the patient developed symptoms of urinary incontinence and gait instability. An MRI scan demonstrated marked ventricular dilatation when compared to his previous images. He was then hospitalized again with a diagnosis of hydrocephalus. After admission, a lumbar puncture showed the initial CSF pressure was 140 mmH_2_O, with positive CSF tap test. A VP shunt was undertaken with an Aesculap Adjustable Valve System (opening pressure 100 mmH_2_O). Correct placement of the ventricular catheter was accomplished at the first attempt. There was no observance of ventricular cannulation produced blood in the cerebrospinal fluid. The postsurgery course was uneventful. The patient did not have a history of hypertension and his blood pressure was within the normal range after surgery. Neither anticoagulation nor antiplatelet therapy was indicated in the perioperative management. A routine CT scan was administered on the 8th day following surgery and showed similar ventricle sizes when compared to the presurgery images (Figure 1A, B). The valve was reprogrammed to 60 mmH_2_O before the patient was discharged the same day.

However, he was sent back to our hospital again 8 h after being home for sudden decrease of consciousness and urinary incontinence. The neurological examination showed that the patient was in a coma with a GCS score of 10. The CT scan revealed a large right frontal intraparenchymal and intraventricular hematoma (Figure 1C, D). Emergency surgery was suggested but was declined by his relatives. Palliative care was then offered and the patient was discharged 3 days later according his relative's decision.

Informed consent was given by relatives of the patient. The institutional review board of the Sir Run Run Shaw Hospital approved the report.

## DISCUSSION

Postshunt intracerebral hemorrhage is one of the complications of VP shunt surgery. It may be caused by puncture of the choroid plexus, repeated attempts at perforation of the ventricles or inadequate placement of the tubing within the parenchyma of the brain.^2^ Delayed hemorrhage is very rare since most bleeding usually occurs in the early stage after surgery. In this instance, we reported a typical case of DIHAV where no blood was observed following ventricle cannulation or during the first CT scan. However, extensive ICH was observed in the CT scan 8 days after surgery. In our institution, it was the only confirmed case of DIHAV in the past 5 years (1/258 VP shunt surgeries).

Previously, Snow et al reported a similar incidence of hemorrhage after ventricular shunting procedures of 0.3%. However, cases with small amounts of blood in the brain without significant symptoms might have been missed if image studies were not performed for uneventful cases. It is therefore not surprising to see that the prevalence of DIHAV was higher in centers where routine postoperative CT or MRI scans were ordered. Savitz et al reported a series of 125 patients operated on for placement of the VP shunting system. Postoperative CT scans were obtained in every case within 48 h of shunt surgery and 5 cases of delayed hemorrhage were recorded.^1^ Similar research performed by Fukamachi et al revealed that 10 out of 242 patients had small intracerebral hematomas as demonstrated by a CT scan within the first week after ventricular shunting procedures. The hemorrhage incidence rate reported in these studies was around 4%.^3^ Nevertheless, it is important to note that both of the series made the diagnosis of DIHAV based on the first CT scan after surgery and most of the hematoma volume was small. Thus, the possibility of minor bleeding occurring immediately after ventricle cannulation cannot be entirely excluded. The true incidence of DIHAV is therefore still difficult to establish.

The mechanism of the DIHAV is controversial. Matsumura et al first reported a case of DIHAV in a 17-year-old patient on the seventh postoperative day in 1986.^4^ The authors attributed this complication to the increased intracranial venous pressure produced by Valsalva's effect. Other possible predisposing risk factors are as follows: (1) mechanical disruption of intravascular vessel by catheter, (2) coagulation disorder, (3) hemorrhage from an occult intravascular malformation or an intracerebral tumor, and (4) head trauma occurring shortly after VP shunt placement. Most reports support the idea that normal pulsations of the CSF transmit to the ventricular catheter that helps the catheter to erode a blood vessel and cause ICH. As shown in Table 1, most hematomas located around the catheter tracts further justify the traumatic injury hypothesis. Mascalchi et al presented a patient with an extensive intraparenchymal hemorrhage 15 days after VP shunt placement. This case was unique because the delayed hemorrhage was located in the contralateral hemisphere of the catheter. The author believed that the decrease of tamponade forces secondary to the sudden reduction of CSF pressure after placement of the VP shunt might cause brain hemorrhage.^5^ Okazaki et al presented an infant with delayed GMH after VP shunt surgery. As the hemorrhage was apart from the catheter tract, they also attributed the delayed bleeding to the reduction of CSF pressure instead of the direct traumatic injury.^6^ In addition, VP shunt-induced disseminated coagulation (DIC) may also be another potential cause. Khandelwal et al presented a rare case of DIC and bilateral thalamic bleeding after VP shunting. They hypothesized that injury to the brain causes the release of thromboplastin into the systemic circulation with the potential development of DIC.^7^

As for our case, the cause of the delayed ICH remains unknown. However, laboratory tests and radiological studies have excluded the possibility of coexistent coagulation disorder (pre-existent bleeding disorder, surgery-induced DIC), occult vascular malformation, and brain tumour or head trauma. Apparently, acute traumatic injury by the catheter may not be the cause since no blood was observed following ventricle cannulation and the first CT scan performed after the shunting procedure did not reveal any hemorrhage either. Also, it is important to note that the patient had a history of brain trauma and subsequent brain surgery. The post-traumatic vasculogenesis around the hypoxic brain tissue and the potential degenerative vasculopathy may have increased the possibility of delayed erosion of the vessel by the catheter. Furthermore, as the patient deteriorated 8 h after downregulation of valve opening pressure, handling of the valve system may be another contributing factor to the late-onset of the hemorrhage. The decreased opening pressure of the valve system can result in a marked reduction of intraparenchymal pressure. This could increase the initially tamponaded vessel disruption and lead to extensive intraparenchymal and intraventricle hemorrhage.

## CONCLUSION

Delayed intracerebral hemorrhage is a rare but severe complication after VP shunt surgery. Erosion of the vasculature by catheter cannulation and a sudden CSF pressure reduction following valve reprogram may be one of the causes of delayed hemorrhage in our case.
